# Gene expression dynamics during the gonocyte to spermatogonia transition and spermatogenesis in the domestic yak

**DOI:** 10.1186/s40104-019-0360-7

**Published:** 2019-07-12

**Authors:** Guowen Wang, Yongchang Li, Qilin Yang, Shangrong Xu, Shike Ma, Rongge Yan, Ruina Zhang, Gongxue Jia, Deqiang Ai, Qi’en Yang

**Affiliations:** 1 Key Laboratory of Adaptation and Evolution of Plateau Biota, Northwest Institute of Plateau Biology, Chinese Academy of Sciences, Xining, 810000 Qinghai China; 2 University of Chinese Academy of Sciences, Beijing, 100049 China; 3 Department of Veterinary Sciences, Qinghai Vocational and Technical Institute of Animal Husbandry and Veterinary, Qinghai University, Xining, 810016 China; 4 Qinghai Academy of Animal Science and Veterinary Medicine, Qinghai University, Xining, 810016 China; 5 Animal Husbandry Technology Extension Station of Qinghai Province, Xining, 810001 Qinghai China; 6 Qinghai Key Laboratory of Animal Ecological Genomics, Northwest Institute of Plateau Biology, Chinese Academy of Sciences, Xining, 810001 Qinghai China; 7 CAS Center for Excellence in Tibetan Plateau Earth Sciences, Chinese Academy of Sciences, Beijing, 100101 China

**Keywords:** Gonocyte, Meiosis, Spermatogenesis, Spermatogonia, Transcriptome

## Abstract

**Background:**

Spermatogenesis is a cellular differentiation process that includes three major events: mitosis of spermatogonia, meiosis of spermatocytes and spermiogenesis. Steady-state spermatogenesis relies on functions of spermatogonial stem cells (SSCs). Establishing and maintaining a foundational SSC pool is essential for continued spermatogenesis in mammals. Currently, our knowledge about SSC and spermatogenesis is severely limited in domestic animals.

**Results:**

In the present study, we examined transcriptomes of testes from domestic yaks at four different stages (3, 5, 8 and 24 months of age) and attempted to identify genes that are associated with key developmental events of spermatogenesis. Histological analyses showed that the most advanced germ cells within seminiferous tubules of testes from 3, 5, 8 and 24 months old yaks were gonocytes, spermatogonia, spermatocytes and elongated spermatids, respectively. RNA-sequencing (RNA-seq) analyses revealed that 11904, 4381 and 2459 genes were differentially expressed during the gonocyte to spermatogonia transition, the mitosis to meiosis transition and the meiosis to post-meiosis transition. Further analyses identified a list of candidate genes than may regulate these important cellular processes. *CXCR4*, a previously identified SSC niche factor in mouse, was one of the up-regulated genes in the 5 months old yak testis. Results of immunohistochemical staining confirmed that CXCR4 was exclusively expressed in gonocytes and a subpopulation of spermatogonia in the yak testis.

**Conclusions:**

Together, these findings demonstrated histological changes of postnatal testis development in the domestic yak. During development of spermatogonial lineage, meiotic and haploid germ cells are supported by dynamic transcriptional regulation of gene expression. Our transcriptomic analyses provided a list of candidate genes that potentially play crucial roles in directing the establishment of SSC and spermatogenesis in yak.

**Electronic supplementary material:**

The online version of this article (10.1186/s40104-019-0360-7) contains supplementary material, which is available to authorized users.

## Background

Spermatogenesis is supported by coordinated gene expressions in germ cells and somatic cells. In mammals, spermatogenesis consists of three phases: mitosis of spermatogonia, meiosis of spermatocytes and spermiogenesis [1]. Spermatogonial stem cells (SSCs) reside within the undifferentiated spermatogonial population and sustain continual spermatogenesis by providing committed progenitors [2, 3]. Progenitor spermatogonia respond to retinoic acid (RA) stimulation and become differentiating spermatogonia, which then expand in number before entering meiosis. After two rounds of meiotic divisions, haploid germ cells are generated and then a unique differentiation process is initiated to produce elongated spermatids [4]. Development of spermatogenic cells are supported by Sertoli cells, Leydig cells, peritubular myoid cells and other somatic cells that reside in the interstitial space of testis [4, 5]. Gene expressions in germ cells and testicular somatic cells therefore must be tightly controlled to ensure normal spermatogenesis and male fertility.

In mammals, the spermatogonial lineage is developed from gonocytes (prospermatogonia). Gonocytes, daughter cells of primordial germ cells, localize to the center of the seminiferous cord and morphologically, each gonocyte have a large, light, spherical nucleus containing two or more globular nucleoli [6]. In the fetal testis, gonocytes proliferate a few days then enter a quiescent state. During the neonatal period of development, gonocytes resume mitosis and follow two different developmental pathways: one group of cells directly becomes differentiating spermatogonia and another group of cells develop to establish the SSC pool [7, 8]. In cattle, gonocytes are localized in the lumen of the seminiferous tubule at birth and they migrate and develop into SSCs and progenitor spermatogonia from 3 to 5 months in *Bos taurus* bulls or 9 to 9.5 months in *Bos indicus* breeds [9, 10]. SSCs maintain their activities under the influence of intrinsic and extrinsic factors. Identifying niche factors and transcription regulators that dictate spermatogonia fate decisions is required for the successful establishment of SSC enriched culture systems [11, 12]. The gonocyte to spermatogonia transition has been intensively studied in genetically engineered mouse and rat models [13, 14], however, molecules directing this crucial event in domestic animals remain to be discovered.

Dissecting molecular mechanisms that direct spermatogonial differentiation, meiosis and spermatid development in domestic animals will enhance our understanding of germ cell development in general and help improve animal reproductive efficiency. Gene expression analyses of testicular tissues from 3 and 9 months old sheep led to the discovery of several candidate genes that play crucial roles in Leydig cell maturation [15]. Transcriptome signatures of testes form Holstein bulls were revealed to dissect gene expression patterns of male specific region of the Y chromosome [16]. Whole testis transcriptome analyses uncovered that genes regulating lipid metabolism were differentially expressed in Large White and Iberian pigs [17]. In addition to mRNA, expression patterns of microRNA, piRNA and long non-coding RNAs were described and functions of these transcripts in the testis development were revealed in several domestic animals [15, 18, 19]. Together, these findings indicated that high-throughput RNA-seq served as a powerful tool to dissect gene expression patterns and identify candidate genes for functional validation in the testis development and spermatogenesis.

The domestic yak (*Bos grunniens*) is the most important livestock on Qinghai-Tibet Plateau because of its ability to adapt to extreme temperature and low oxygen environment. Reproductive efficiency of the yak is low under the traditional grazing system and male yaks reach sexually maturity at 3 or 4 years after birth. Recently, the testes transcriptome data of the yak and its hybrid offspring cattle-yak were generated to identify genes that may play significant roles in hybrid male sterility [20]. However, development of the spermatogonial population and particularly, meiotic and post-meiotic spermatogenic cells have not been examined and candidate genes directing germ cells fate decisions at different postnatal developmental stages remain to be uncovered in the yak. In the present study, we examined histology and gene expression dynamics during the gonocyte to spermatogonia transition and spermatogenesis in the domestic yak. The aim of this study was to decipher cellular differentiation processes during postnatal germ cell development and identify candidate genes that direct three major phases of spermatogenesis: establishment of the spermatogonial lineage, meiosis and spermiogenesis.

## Methods

### Animals

Animals were raised at the Datong yak farm under grazing conditions from June to August. Male yaks of 3, 5, 8 and 24 months old were castrated using a standard protocol [21] and testicular tissues were processed for RNA extraction or histology.

### Tissue collection and processing

Testes were removed immediately after castration and crosscut into the size of 5 mm × 5 mm and fixed in 4% paraformaldehyde (PFA) or Bouin’s solution for 12 h at 4 °C. Tissues were dehydrated in 30%, 50%, 75%, 95% and 100% ethanol, and then treated with xylenes before embedding in paraffin. For RNA extraction, tissues were cut into small pieces and snap-frozen in liquid nitrogen immediately and then stored at − 80 °C.

### Histology and immunohistochemistry

To examine histology, testicular tissues that were fixed in Bouin’s solution were sectioned and 5 μm sections were deparaffinized and rehydrated as described previously [22]. The sections were washed in PBS twice and stained with hematoxylin and eosin (H&E). To conduct immunohistochemical staining, testicular tissues that were fixed in 4% PFA were used. Briefly, rehydrated slides were boiled in 10 mmol/L trisodium citrate (pH 6.0) in a microwave oven for 20 min for antigen retrieval. The endogenous peroxidase activity was blocked by using 3% H_2_O_2_ for 10 min at room temperature (RT) and washed with PBS. After blocking with 10% serum in PBS for 1 h at RT, the slides were then incubated with primary antibodies overnight at 4 °C. Sections were washed with PBS and incubated with HRP-conjugated secondary antibody. The immunoreactive signal was visualized by applying the 3,3-diaminobenzidine (DAB, ZSGB-BIO, Beijing, China) and counterstained with hematoxylin. Images were examined under a microscope (Nikon ECLIPSE E200), and digital images were captured (MshOt MS60).

To conduct PNA staining, the rehydrated slides were boiled in a microwave oven for 20 min and washed with PBS three times. Subsequently, the slides were incubated with a primary antibody with at 4 °C overnight, and then washed with PBS. After counter staining DNA with H33342 (Sigma, 20 μg/mL) for 30 s, the slides were covered with glycerine and examined under a microscope (Leica, Germany).

The following antibodies were used in the study: anti-SYCP3 (Abcam, USA, rabbit polyclonal antibody (pAb);1:500 dilution, 0.2 μg/mL),anti-PLZF (Santa Cruze, USA, rabbit pAb;1:200 dilution, 2.5 μg/mL), anti-CXCR4 (Santa Cruze, USA, rabbit pAb;1:50 dilution, 10 μg/mL), anti-KI67 (Abcam, USA, 1:400, 2.5 μg/mL), anti-PNA (Vector Labs,1:500 dilution), HRP conjugated goat anti-rabbit IgG (Ruiying Bio, China, 1:200 dilution, 20 μg/mL).

### RNA extraction, library construction and sequencing

For each age group, tissues from 3 animals were used for RNA extraction, library construction and sequencing. Briefly, total cellular RNA of each yak testis was isolated using the Trizol Kit (Promega, USA) following the manufacturer’s instructions. RNA quality and concentration were measured by using Agilent 2100 Bio-analyzer (Agilent Technologies, Santa Clara, CA) and by RNase free agarose gel electrophoresis after removing the genomic DNA. Next, Poly (A) mRNA was isolated using oligo-dT beads (Qiagen). All mRNA was broken into short fragments by adding fragmentation buffer. First-strand cDNA was generated using random hexamer-primed reverse transcription, followed by the synthesis of the second-strand cDNA using RNase H and DNA polymerase I. The cDNA fragments were purified using a QIA quick PCR extraction kit. These purified fragments were then washed with EB buffer for end reparation poly (A) addition and ligated to sequencing adaptors. Following agarose gel electrophoresis and extraction of cDNA from gels, the cDNA fragments were purified and enriched by PCR to construct the final cDNA library. The cDNA library was sequenced on the Illumina sequencing platform (Illumina HiSeq™ 2500) using the paired-end technology by Gene Denovo Co. (Guangzhou, China). A Perl program was written to select clean reads by removing low quality sequences (there were more than 50% bases with quality lower than 20 in one sequence), reads with more than 5% N bases (bases unknown) and reads containing adaptor sequences.

### Transcript assembly and expression value estimation

BosGRu_v2.0 was used for alignment of RNA sequencing reads. Sequencing reads in FASTQ format were mapped to reference genome as well as splice junctions were identified using TopHat [23]. Cufflinks package [24] was used to genome guided transcript assembly and expression abundance estimated. First, Cufflinks was used to reconstruct transcript based on genome annotation, then the transcripts from each sample were merged by cuffmerge. Novel transcripts were extracted from the result with threshold of “length ≥200 bp and exon number ≥” , and they were compared with 3 protein databases to obtain function annotation using blastx [25] with E-value cut-off of 1e-5. The databases contained NCBI non-redundant protein database (Nr) (http://www.ncbi.nlm.nih.gov/), KEGG (http://www.kegg.jp/) and GO (http://geneontology.org/). Next, the novel transcripts were integrated with the existing transcript in genome annotation to construct a new gft file. Finally, cuffquant and cuffnorm were used to estimate transcript expression value as FPKM with parameter library normalization methods: classic-fpkm, library types: fr-unstranded.

### Cluster analysis

DAVID database was used for functional annotation (https://david.ncifcrf.gov/). The significantly changed gene IDs of the RNA-seq data were converted to the IDs consistent with DAVID. Converted IDs were loaded to DAVID listing KEGG pathways and functional clusters.

### Statistical analysis

To quantify PLZF, SYCP3 or Ki67 positive cells, at least 100 round seminiferous tubules from 5 serial sections of each animal were examined. Three animals were used in the experiment. All the statistical data were presented as means ± SEM for three biological replicates. Differences between means were examined using the One-way ANOVA of GraphPad Prism5 (La Jolla, CA, USA). Differences between means were considered significant at *P* < 0.05.

## Results

### Histological analysis of spermatogenic cell development in postnatal yak testis

**Fig. 1:**
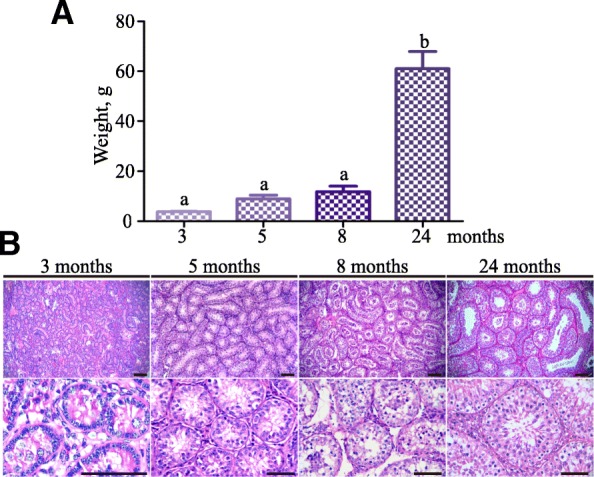
Histological analyses of the yak testis development at four different developmental stages. **a** Testes weight of 3, 5, 8 and 24 months old domestic yaks. **b** H&E staining of testes from 3, 5, 8, 24 months old yaks. Data are presented as mean ± SEM of at least four yaks in **a**. Different letter denotes significantly different at *P* < 0.05. Scale bar = 50 μm.

We conducted histological analysis to examine morphological changes of spermatogenic cells during postnatal development in the yak. Testes were collected from 3, 5, 8 and 24 months old yak and testicular sections were stained with hematoxylin and eosin (H&E). Testis weight was increased significantly at 24 months (Fig. 1a). At 3 months, germ cells were observed in the center of seminiferous cords and morphology of the cells was similar to gonocytes of the neonatal mouse testis (Fig. 1b). At 5 months, gonocyte migrated and differentiated into spermatogonium, which contained a large nucleus with limited chromatin in the nuclear envelop as described previously [26] (Fig. 1b). At 8 months, meiotic cells appeared and some seminiferous tubules contained round spermatids (Fig. 1b). As expected, seminiferous tubules of 24 months testis contained all types of spermatogenic cells: spermatogonia, spermatocytes, round and elongated spermatids (Fig. 1b). Together, these data demonstrated that testicular samples examined in the present study covered three crucial events during postnatal germ cell development in yak.

### Immunohistological analyses of spermatogonia, meiotic and post-meiotic germ cell markers in yak testis

**Fig. 2:**
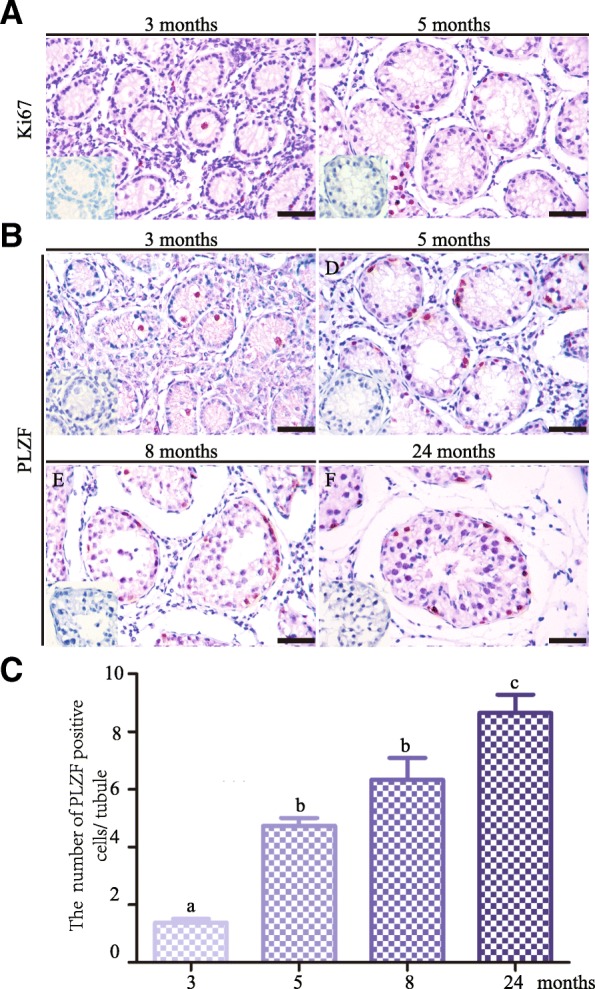
Immunohistological analyses of germ cell differentiation in the yak testis at four different developmental stages. **a** Immunostaining for Ki67 in testicular sections from yaks at 3 and 5 months. **b** Immunostaining for PLZF in testicular sections from yaks at 3, 5, 8 and 24 months. **c** Quantification of PLZF^+^ cells per seminiferous tubules at four different developmental stages. Negative control was shown in the lower-left corner. Different letter denotes significantly different at *P* < 0.05. Scale bar = 50 μm

Next, we conducted marked spermatogonia, spermatocytes and spermatids using immunohistology to confirm the results of histological analyses. Firstly, we found that all gonocytes were positive for PLZF (550 gonocytes from 3 animals) and were localized in the center of seminiferous tubules, indicating that gonocytes did not initiate migration at 3 months. Interestingly, 30.8% ± 1.5% of germ cells were positive for Ki67, while all Sertoli cells were negative for Ki67, which is expressed in proliferative cells (Fig. 2a), suggesting that germ cells already reentered mitotic cell cycle and Sertoli cells already entered quiescence. All PLZF^+^ cells were distributed on the basement membrane and were surrounded by Sertoli cells at 5 months, indicating gonocytes completed migration and became spermatogonia (Fig. 2b). PLZF^+^ cells per seminiferous tubule were 1.4 ± 0.6 at 3 months, 4.7 ± 1.3 at 5 months, 6.3 ± 1.9 at 8 months, and 8.6 ± 2.4 at 24 months, respectively (Fig. 2c). These data indicated that the gonocyte to spermatogonia transition occurred from 3 to 5 months in the domestic yak.

**Fig. 3:**
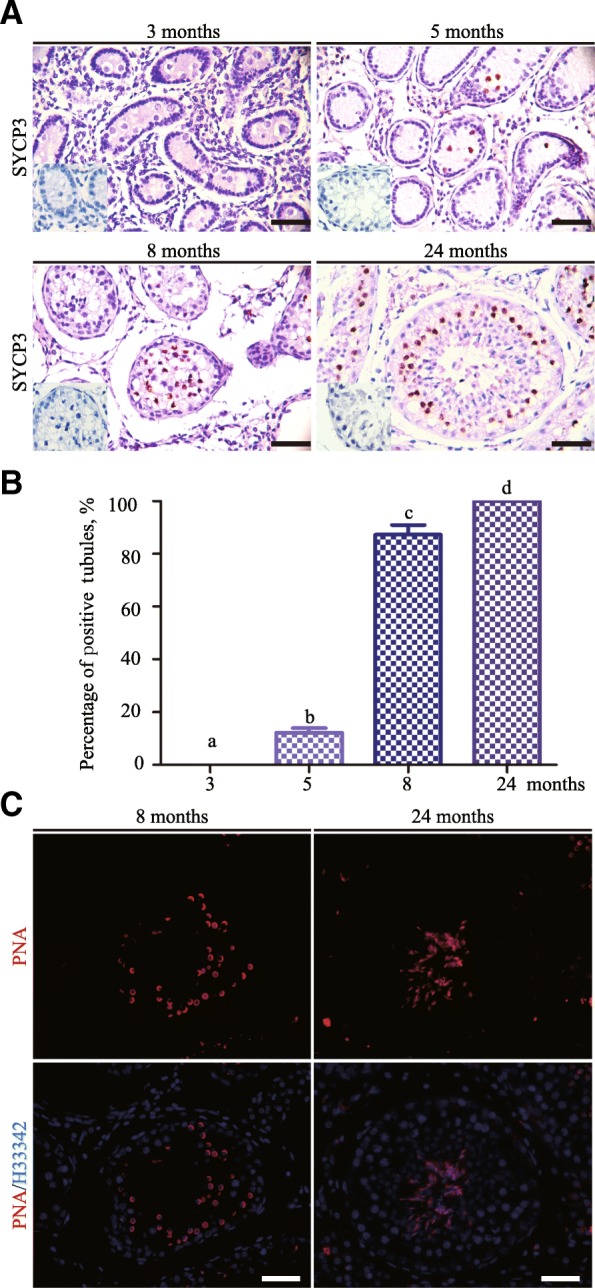
Development of meiotic and post-meiotic germ cells in the yak testis **a** Immunostaining for SYCP3 in testicular sections from yak at 3, 5, 8, and 24 months. Negative control was shown in the lower-left corner. **b** Percent of seminiferous tubules containing spermatocytes in testes of 3, 5, 8, 24 months old yaks. **c** PNA staining of seminiferous tubules from 8 and 24 months old yak testes, nuclei was counterstained with Hoechst 33342. Different letter denotes significantly different at *P* < 0.05. Scale bar = 50 μm

Next, we found that germ cells were negative for SYCP3 at 3 months (Fig. 3a). However, at 5 months, 12.2 %± 4.0%of seminiferous tubules contained SYCP3^+^ cells (Fig. 3b), suggesting small number of preleptotene spermatocytes already appeared. At 8 months, 87.3% ± 8.2% of seminiferous tubules contained SYCP3^+^ cells (Fig. 3b). Furthermore, round spermatids began to emerge as indicated by PNA staining, which recognizes acrosomal lectins in spermatids (Fig. 3c). These findings indicated that development of meiotic and post-meiotic germ cells occurred from 5 to 8 months. At 24 months old bull testis, spermatogonia, spermatocytes (Fig. 3a), round and elongated spermatids (Fig. 3c) were all present, representing steady-state spermatogenesis. Together, immunohistological results confirmed the histological observations that the samples examined in the present study covered crucial development periods of spermatogenesis in the yak.

### Transcriptomic profiling of yak testes at different developmental stages

**Fig. 4:**
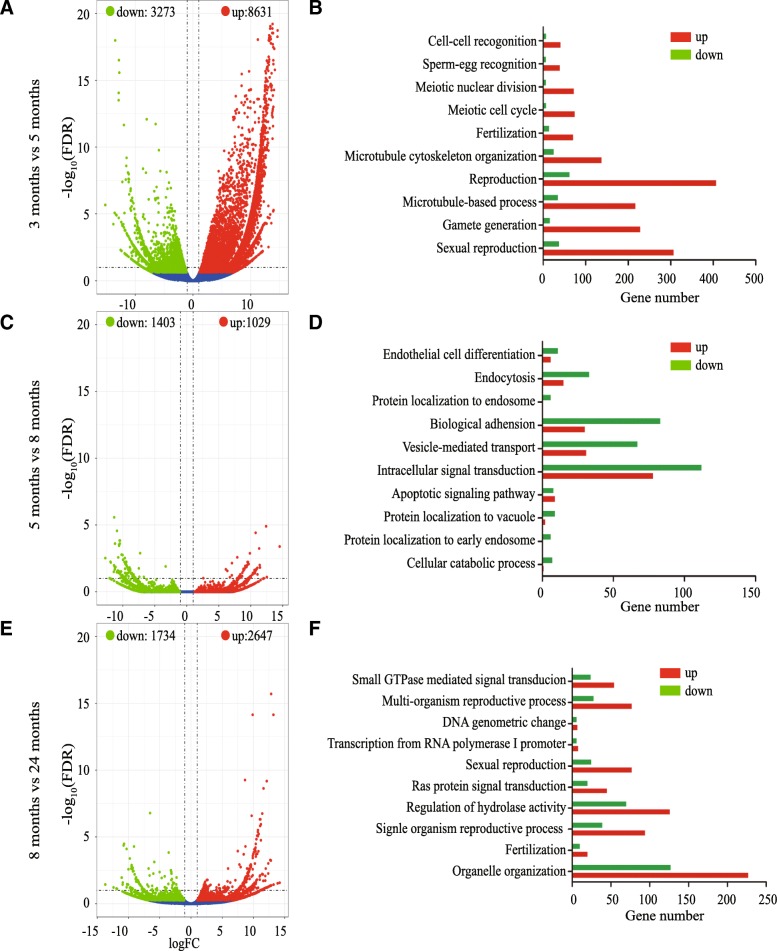
Transcriptional profiling and gene to ontology of yak testes at different developmental stages. **a** Volcano plot of testicular transcriptome from 3 and 5 months old yak (*n* = 3 each); **b** Volcano plot of testicular transcriptomes of 5 and 8 months testes (*n* = 3 each); **c** Volcano plot of testicular transcriptome of 8 and 24 months testes (*n* = 3 each). **d** Gene ontology analyses of up-regulated and down-regulated genes from 3 and 5 months testes. **e** Gene ontology enrichment of up regulated and down regulated genes in testis from 5 and 8 months testes. **f** Gene ontology enrichment of up regulated and down regulated genes of testis from 8 and 24 months testes. Red denotes up-regulated genes; green denotes down-regulated genes, blue denotes genes that did not show significant difference

After recognizing the differentiation processes of germ cells in the postnatal yak testis, next we conducted transcriptomic profiling of testes from 3, 5, 8, and 24 months old yak using RNA-seq. In total, we acquired 52810358, 47718406, 46742091, and 52575007 clean reads from testis at the four stages, respectively. Of 85.47%, 89.53%, 84.30%, and 89.63% clean reads were mapped to reference genome, and 50534, 50823, 52068 and 49634 genes were annotated. By conducting global gene expression analysis, we found that 11904 genes were significantly expressed between 3 and 5 months old testis (Fig. 4a, and Additional file 1: Table S1). Specifically, 8431 genes were up regulated and, 3273 were down regulated (Fig. 4a). Interestingly, when the mitosis to meiosis transition occurred from 5 to 8 months, 2647 genes were up-regulated and 1734 were down-regulated (Fig. 4b and Additional file 2: Table S2). The total number of differentially expressed genes was decreased when round spermatids became elongating spermatid and somatic cells matured from 8 months to 24 months, because only 1029 genes were up-regulated and 1403 were down-regulated in 24 months (Fig. 4c, Additional file 3: Table S3).

### Gene expression patterns during the gonocyte to spermatogonia transition

**Fig. 5:**
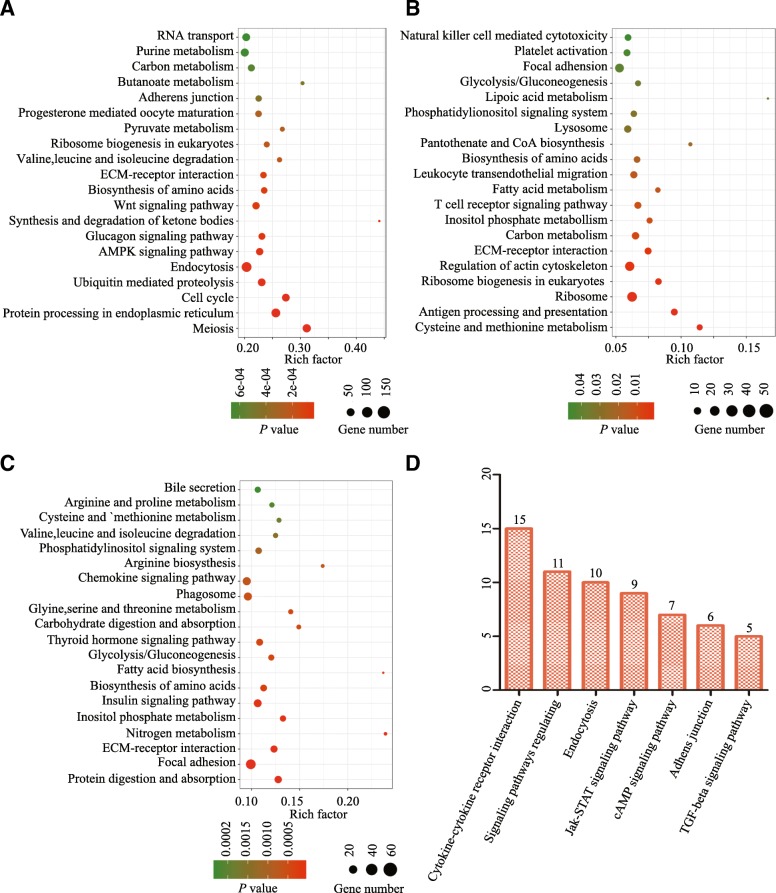
KEGG and gene ontology pathway analyses of selected genes in testes of 5 months old yaks. **a** Top 20 KEGG pathways of differential expressed genes are listed between 3 and 5 months old yak testes. **b** Top 20 KEGG pathways of differential expressed genes are listed between 5 and 8 months old yak testes. **c** Top 20 KEGG pathways of differential expressed genes are listed between 8 and 24 months old yak testes. KEGG pathways are listed by *P* value. *P* < 0.05 for all pathways. **d** Pathways of up-regulated genes encoding receptors at 5 months compared with those of 3 months yak testes

Next, we conducted Gene Ontology (GO) and KEGG analyses to further dissect gene expression dynamics underlying the gonocyte to spermatogonia transition. GO analysis showed prominent ontological cluster of genes associated with sexual reproduction, gamete generation, meiotic cell cycle and meiotic nuclear division (Fig. 4d). Top 10 pathways for both up regulated and down included meiosis, protein processing in endoplasmic reticulum, ubiquitin mediated proteolysis, endocytosis, AMPK signaling pathway, glucagon signaling pathway, Wnt signaling pathway, (Fig. 5a). Because during this period of development, gonocytes resumed mitosis and migrated to the basement membrane, it was not surprising that genes control cell adhesion were differentially expressed. Integrin-mediated cell adhesion to the extracellular matrix (ECM) was essential for maintaining the stem-cell niche [27] and *ITGB1, ITGB4, ITGB8, ITGA2B,ITGAV, ITGA4, ITGA7* and *ITGA9* were significantly up regulated in the testis of 5 months old yak. Surprisingly, however, transcripts encode proteins regulating meiosis progression *(SYCP1*, *SYCP2*, *SYCP3*, *SPO11*, *BRCA1*, *TEX15*, *MOV10L1*, *TDRD1*, *HORMAD1*, and *HORMAD2*) were already up-regulated at this stage.

### Gene expression patterns during spermatogonial differentiation, meiosis and spermiogenesis

From 5 to 8 months, GO terms were: biological adhesion, endocytosis, intracellular signal transduction (Fig. 4e). Top 10 KEGG pathways of the differentially expressed genes primarily were associated with cysteine and methionine metabolism, antigen processing and presentation, ribosome, ribosome biogenesis in eukaryotes, regulation of actin cytoskeleton, ECM-receptor interaction, carbon metabolism, inositol phosphate metabolism, T cell receptor signaling pathway, fatty acid metabolism (Fig. 5b). From 8 to 24 months, GO terms were: reproductive process, organelle organization, DNA geometric change and small GTPase mediated signal transduction (Fig. 4f). Top 10 KEGG pathways were: protein digestion and absorption, focal adhesion, ECM-receptor interaction, nitrogen metabolism, inositol phosphate metabolism, insulin signaling pathway, biosynthesis of amino acids, fatty acid biosynthesis, gluconeogenesis, thyroid hormone signaling pathway (Fig. 5c). It was worth noting that steroid metabolic process was significantly up-regulated from 8 to 24 months, likely because Leydig cells were functionally matured and the synthesis and metabolism of testosterone were tightly controlled to support the development of spermatids. For example, *CYP7B1*, SRD5A1, *HSD17B1*, *HSD17B4*, *HSDL2* were up-regulated by 19.5, 5.3, 3.4, 1156.6, 14203.3 folds, respectively.

### Expression profiling of membrane and nuclear receptors in yak testis

In testis of 5 months old yak, undifferentiated spermatogonia were enriched and a functional SSC pool must be established to sustain continual spermatogenesis. We attempted to identify ligand-receptor signaling that might direct spermatogonial fate decisions and to reveal potential markers that label undifferentiated spermatogonia in the yak. We screened cell surface and nuclear receptors and uncovered a list of receptors that were up-regulated in the testis of 5 months old yak compared with those in the testis of 3 months old yak (Table 1). Interestingly, expression levels of receptors for previously identified niche factors Glial cell line-derived neurotrophic factor (GDNF), and epidermal growth factor (EGF) were not changed [2, 28]. Expression of Thy1, which marks cells containing enriched SSC activities in bull, goat and boar [29–31], was not increased at transcription level. We identified 299 up-regulated receptors and the KEGG analysis (Fig. 5d) suggested that cytokine-cytokine receptor interaction, signaling pathways regulating pluripotency of stem cells, Jak-STAT signaling pathway, adhesion junction, endocytosis, TGF-beta signaling pathway, cAMP signaling pathway were significantly enriched. Among the receptors, we found that CXCR4 expression was increased more than 13400 folds and our previous study revealed a crucial role of CXCR4-dependent signaling in maintaining the undifferentiated spermatogonia population in mice. A recent study revealed that CXCR4 positive germ cells may contained enriched SSC activities [32].

**Table 1:** List of up regulated genes in the 5 months old yak testis

SA	Description	Fold change	*P* value	*q* value
*KDELR2*	ER lumen protein retaining receptor 2	30,060.05	1.10E-10	1.23E-08
*CXCR4*	C-X-C chemokine receptor type 4	13,403.33	1.02E-08	6.70E-07
*PTPN18*	Tyrosine-protein phosphatase non-receptor	12,003.35	6.26E-05	0.001343
*ITGB1*	Integrin beta-1	11,693.34	0.000603	0.008664
*ADORA3*	Adenosine A3 receptor	10,380.01	8.32E-09	5.71E-07
*LBR*	Lamin-B receptor	5266.65	1.20E-07	5.81E-06
*PTPN13*	Tyrosine-protein phosphatase non-receptor type 13	5190.004	1.72E-06	6.06E-05
*PTPN11*	Tyrosine-protein phosphatase non-receptor type 11	5079.99	2.19E-07	9.85E-06
*RARB*	Retinoic acid receptor beta	3736.677	8.52E-05	0.001734
*BMPR2*	Bone morphogenetic protein receptor type-2	3263.332	3.52E-08	1.95E-06
*FLT1*	Vascular endothelial growth factor receptor 1	3153.329	7.03E-09	4.92E-07
*BMPR1A*	Bone morphogenetic protein receptor type-1A	3089.998	1.23E-05	0.000335
*ACVR2A*	Activin receptor type-2A precursor	2266.672	0.001563	0.023021
*OLR1*	Oxidized low density lipoprotein (lectin-like) receptor 1	2233.329	0.000398	8.84E-07
*PTPN4*	Tyrosine-protein phosphatase non-receptor type 4	1953.33	0.000263	1.95E-06
*FGFR1*	Fibroblast growth factor receptor 1	1936.667	0.064243	0.011071
*IL13RA2*	Interleukin-13 receptor subunit alpha-2	1719.997	0.009357	4.92E-07
*TLR3*	Toll-like receptor 3	1293.335	0.017564	0.000335
*GHR*	Growth hormone receptor	1206.665	0.002133	0.000461
*EPCAM*	Epithelial cell adhesion molecule	6.298398	0.000666	0.009357
*CD9*	CD9 antigen	3.554082	0.300861	0.640542
*THY1*	Thy-1 cell surface antigen	0.497514	0.15409	0.456851
*ITGA6*	Integrin alpha 6	0.298429	0.011942	0.087918
*GFRA1*	GDNF family receptor alpha 1	0.122892	0.008254	0.066507

### CXCR4 labels gonocyte and a subpopuation of spermatogonia in yak testis

**Fig. 6:**
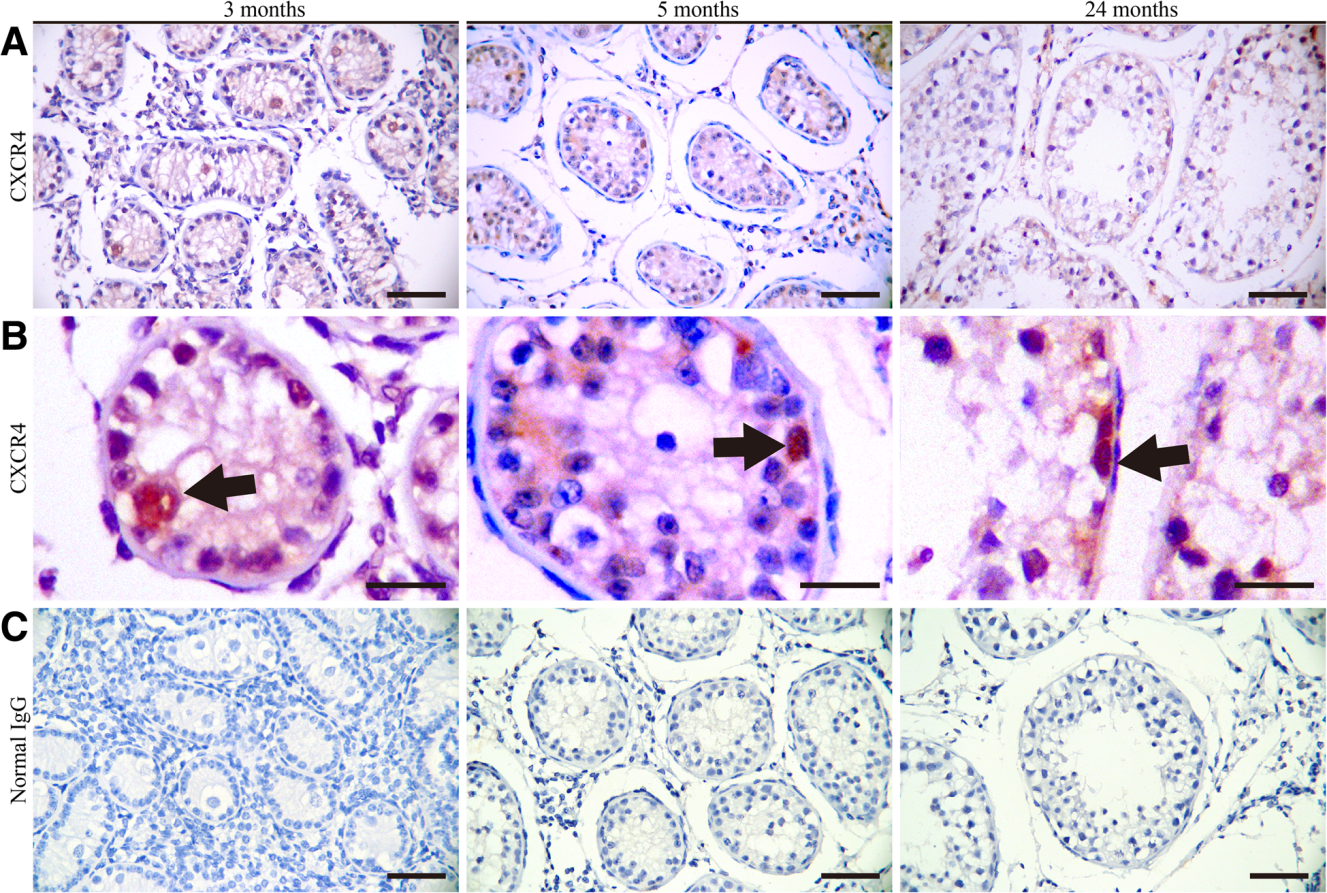
CXCR4 was expressed in gonocytes and a subpopulation of spermatogonia in yak. **a-b** Immunostaining for CXCR4 in testicular sections from 3, 5, and 24 months old yaks. Black arrowheads indicate CXCR4 positive cells. **c** Negative control of each time point. Scale bars represent 50 μm

To further validate this finding, we conducted immunostaining of cross-sections using a CXCR4 specific antibody to identify the expression of CXCR4 in the postnatal yak testis. In testis of 3 months old calf, CXCR4 staining was only seen in gonocytes and it appeared that all gonocytes expressed CXCR4 (Fig. 6a). In the testis of 5 months old yak, a subpopulation of spermatogonia was stained positive for CXCR4 and the number of CXCR4^+^ spermatogonia was rare because only 1.2 ± 0.2 cells were found per seminiferous tubule (Fig. 6b). In the testis of 24 months old yak bull, CXCR4 expression was also restricted to a small number of spermatogonia (Fig. 6a and b). These findings suggested that CXCR4 was specifically expressed in gonocytes and a subtype of spermatogonia. Normal IgG was used as a negative control for all immunohistological staining (Fig. 6c).

## Discussion

In the present study, we examined spermatogenic cell development and global gene expression in postnatal yak testis. The findings revealed that the gonocyte to spermatogonia transition occurred in testis of 3 to 5 months old yak and round spermatid was formed in testis of 8 months old yak, complete spermatogenesis was established in testis of 24 months old yak. We provided the first transcriptomic landscape of yak testis at four different developmental stages and revealed the gene expression dynamics at three period of postnatal germ cell development. Data reported in the present study provided key information for understanding germ cell development in the yak and served as an important source for screening germ cell intrinsic and extrinsic factors that regulate SSC fate decisions, meiosis progression and spermiogenesis. These findings will also serve as a key database to identify misexpressed genes in hybrid sterility between yak and cattle.

Sex maturation is delayed in yak compare with that in Holstein bulls; however, timing of germ cell development is similar between the *Bos taurus* breed and the domestic yak. It is generally accepted that *Bos indicus* bulls and buffalo bulls reach sexual maturity at a later age than *Bos taurus* males, even when both are raised under similar conditions. In Holstein, the spermatogonial population emerges from 3 to 5 months and elongated spermatids were formed from 7 to 8 months [10]. This is in sharp contrast to *Bos indicus* bulls, it was reported that the spermatogonial population was established around 9 to 9.5 months in *Bos indicus* breeds [9, 10]. In Murrah buffalo bulls, gonocytes migration do not reach the basement membrane until 18 months, and elongated spermatids appear at 24 months [33]. We examined age matched yaks and observed that migration of gonocytes to the basement membrane and development of spermatogonia were completed in testis of 5 months old yaks. From the present findings, we concluded that gonocytes differentiation and onset of spermatogenesis is were comparable between *Bos taurus* and *Bos grunniens* bulls.

Testis development from 3 to 5 months was directed by dynamic gene expression in yak. By comparing the number of DEGs, it was clear that among three phases of spermatogenic cell development, the gonocyte to spermatogonia transition was supported by massive changes of gene expression. In mouse, a similar pattern of gene expression was reported [34]. During this period of development, Sertoli cells began to enter quiescence while gonocytes resumed mitosis and migrated to the basement membrane [13]. In yak, Sertoli cells were Ki67 negative at 3 months and stayed quiescent at 5 months, these data suggested that major change of gene expression might occur in germ cells. From KEGG analysis, we found that ubiquitin mediated proteolysis was significantly regulated. When mouse gonocytes differentiate into spermatogonia, ubiquitin-activating enzymes and ligases were decreased and ubiquitin conjugating and deubiquitinating enzymes were increased [35]. Gonocytes are round cells containing large nuclei and undifferentiated spermatogonia are oval shaped cells with a smaller nuclear diameter [36]. Changes of ubiquitin expression and function may be associated with extensive cellular remodeling during gonocytes differentiation. Genes regulating cell adhesion and migration were also differentially expressed. Functional roles of these genes need to be studied using in vitro culture systems.

Because whole testis tissue was used in this study, it was difficult to dissect tissue specific gene expression, however, by comparing the expression patterns of conserved germ cell specific genes [37], we found that majority of genes up-regulated at 5 months are associated with meiosis. We speculated that differentiating spermatogonia was already formed and transcripts encode proteins that control meiosis were transcribed at this developmental stage. At 8 months, when spermatocytes migrated and engaged with Sertoli cells to form blood testis barrier (BTB) [38, 39], genes regulating actin cytoskeleton were significantly changed. When elongating spermatid was found in the testis at 24 months, genes regulating organelle organization and DNA geometric change were differentially expressed. During this period of development, histone to protamine exchange occurs and spermatozoa with acrosome and flagellum forms [40]. Together, the DEG discovered in the present study will provide a valuable reference to screen functionally important genes regulating spermatogonial differentiation, meiosis and spermiogenesis in yak and closely related species.

One of crucial factors identified by analyzing the expression data was CXCR4. By examining the expression pattern of receptors in yak testis during the gonocyte to spermatogonia transition, we uncovered a list of transcripts that were greatly increased in testis of 5 months old yak. Because undifferentiated spermatogonia were enriched at this stage, we hypothesized that these factors may be expressed exclusively in this germ cell subpopulation. Among them were *FGFR1*, *FGFR2*, *LIFR,* and *CXCR4,* receptors for previously identified SSC niche factors in mouse [22, 41, 42]. Previously, we described an important role of CXCR4 in mouse SSC maintenance [22]. Interestingly, recent studies reported that CXCR4^+^ cells contained enriched SSC activity in bull, boar and human testis [32, 43, 44] . Findings from the current study demonstrated that CXCR4 was only expressed in gonocytes and a small number of spermatogonia; we therefore proposed that CXCR4 can be used as a marker for SSC enrichment. Activation of the CXCL12-CXCR4 axis promotes primordial germ cell migration and SSC maintenance [22, 45], whether activation of CXCR4 signaling affects gonocyte migration and SSC fate decision waits further validation in large animals.

## Conclusions

**Fig. 7:**
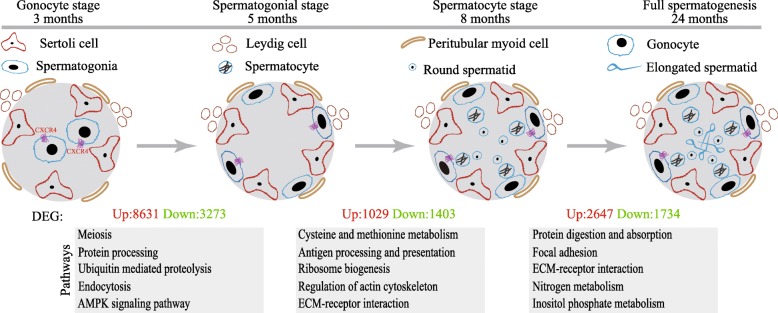
Schematic diagram of postnatal germ cell development and gene expression dynamics in the yak. Histological, immunohistochemical and RNA-seq analyses revealed pathways and candidate genes than may regulate these important cellular processes: the gonocyte to spermatogonia transition, the mitosis to meiosis transition and the meiosis to post-meiosis transition

Together, these findings revealed that the gonocyte to spermatogonia transition and development of meiotic and haploid germ cells in yak are supported by dramatic transcriptional regulation of gene expression (Fig. 7). Our transcriptomic analyses provided a list of candidate genes that play crucial roles in directing the establishment of SSC and spermatogenesis in yak. By further analysis, we provided evidence that CXCR4 was exclusively expressed in gonocyte and a subpopulation of spermatogonia in the yak.

## Additional files

Additional file 1: **Table S1.** Comparative analysis of transcriptomes between 3 and 5 months old yak testes. (XLSX 3910 kb)

Additional file 2: **Table S2.** Comparative analysis of transcriptomes between 5 and 8 months old yak testes. (XLSX 989 kb)

Additional file 3: **Table S3.** Comparative analysis of transcriptomes between 8 and 24 months old yak testes. (XLSX 1600 kb)

## Abbreviations

CXCL12: C-X-C motif chemokine 12
CXCR4: C-X-C chemokine receptor type 4
EGF: Epidermal growth factor
FGFR: Fibroblast growth factor receptor
GDNF: Glial cell line-derived neurotrophic factor
LIFR: Leukemia inhibitory factor receptor
PLZF: Promyelocytic leukemia zinc finger
PNA: Peanut agglutinin
SSC: Spermatogonial stem cell
SYCP3: Synaptonemal complex protein 3
